# A Nationwide Review of the Utilization of Rapid Antigen Testing for Group A Streptococcus

**DOI:** 10.7759/cureus.110915

**Published:** 2026-06-15

**Authors:** Rachel Hammett, Sophie Hackenbruch, Ruth Farrugia

**Affiliations:** 1 Paediatrics and Child Health, Mater Dei Hospital, Msida, MLT

**Keywords:** group a streptococcal pharyngitis, invasive group a streptococcus, paediatric patient, rapid antigen detection test, rapid antigen test, streptococcal pharyngitis, streptococcus group a infection

## Abstract

Background

Group A streptococcus (GAS) is the leading cause of bacterial pharyngitis in children and adolescents, accounting for up to 40% of all cases of pharyngitis in children aged 5 to 15 years. The global incidence of GAS pharyngitis and invasive GAS infections has increased over the past few years. In Malta, Mater Dei Hospital houses the only dedicated pediatric emergency department and is also the only health care facility offering free GAS rapid antigen detection tests (RADTs). As per hospital protocol, the Centor criteria are used to assess the likelihood of GAS pharyngitis.

Method

Staff members in the Pediatric Emergency Department at Mater Dei Hospital in Malta were asked to document all patients who needed a GAS RADT between June 2024 and January 2025. Data were then collected retrospectively from patient records, including demographics, clinical features, antibiotic choice, Centor scores, and RADT results. Analysis was conducted using summative and descriptive statistics, and chi-squared tests to assess associations between Centor score, RADT results, and age groups. A p-value of <0.05 was deemed statistically significant.

Results

A total of 103 patients were included, with a slight male preponderance (55%, N=57). Documentation of Centor criteria was poor, with no documentation in 87% (N=90) of cases. No confirmatory bacterial swabs were taken following a positive rapid antigen swab. All patients with a positive test result received antibiotics, with 88% (N=23) receiving a 10-day course of co-amoxiclav. A positive result was more likely in children over three years of age.

Conclusion

Although RADTs for GAS are readily available for use and positive results prompt appropriate antibiotic prescribing, this study highlights that there are ongoing areas for improvement in the application of clinical criteria, indicating the need for testing and in antimicrobial stewardship. The overuse of RADT and the prescription of antibiotics for children whose RADT is negative emphasize the need for better documentation of the Centor score, improved adherence to the testing algorithm (including Centor score), and the use of culture confirmation for patients with a high clinical suspicion of GAS pharyngitis in spite of negative RADT.

## Introduction

Group A streptococcus (GAS) is the leading cause of bacterial pharyngitis in children and adolescents, accounting for up to 40% of all cases of pharyngitis in children aged five to 15 years [1,2]. The global incidence of GAS pharyngitis and invasive GAS infections has increased in recent years [3].

Timely antibiotic treatment for GAS pharyngitis shortens symptom duration and severity, reduces transmission, and prevents suppurative and non-suppurative complications, including invasive infections and rheumatic fever [4]. Other complications include post-streptococcal glomerulonephritis, post-streptococcal reactive arthritis, and pediatric autoimmune neuropsychiatric disorders associated with Streptococcal infections (PANDAS). In the absence of a specific sign or symptom that can reliably distinguish GAS from viral pharyngitis [5], positive microbiological tests are often used to confirm diagnosis and prevent unnecessary antibiotic treatment. Throat swab culture is the diagnostic gold standard but takes 48 hours to receive a result, while rapid antigen detection tests (RADTs) provide immediate results and have high diagnostic accuracy, with pooled sensitivity and specificity of 85.6% and 95.4%, respectively [1].

In Malta, Mater Dei Hospital houses the only dedicated pediatric emergency department and is also the only health care facility offering GAS RADTs at no cost, as it is the national health service offered to tax payer for free. As per hospital protocol, the Centor criteria [6-8] are used to assess the likelihood of GAS pharyngitis [9]. RADT is recommended for patients aged three to sixteen years with a Centor score ≥3, those with clinical features of scarlet fever, symptomatic close contacts of confirmed GAS cases, and children under three years old with school-aged siblings and presenting with suspected GAS pharyngitis. The Centor criteria are fever, absence of cough, tender cervical lymphadenopathy, and tonsillar exudate. Patients with positive RADT results should then be treated with a 10-day course of penicillin V or amoxicillin, in line with international guidelines [10].

The aim of this study was to evaluate the appropriateness of GAS RADT use at Mater Dei Hospital in the pediatric population, focusing on adherence to Centor criteria and subsequent prescribing practices. 

This article was previously presented as a meeting poster at the 11th Malta Medical School Conference 2025 on 5th December 2025.

## Materials and methods

Staff members in the Pediatric Emergency Department at Mater Dei Hospital in Malta were asked to document all patients who needed a GAS RADT between June 2024 and January 2025. Swabbing was performed by the attending physician when clinically indicated, as per Centor criteria. The RADT test in use was the Test IT kit from Türklab.

Data were then collected retrospectively from patient records, including demographics, clinical features, antibiotic choice, Centor scores, and RADT results. Cases were identified by relying on colleagues to document which patients had RADT testing. In addition, all patient records for each day were checked to avoid missing data. Data were anonymized prior to analysis. Authorization was obtained from the Chair of the Department of Child and Adolescent Health and the Data Protection Officer. Ethical approval was not required as no direct patient contact occurred.

All children below the age of 16 were eligible for inclusion, irrespective of their swab result. Analysis was conducted using summative and descriptive statistics, and chi-square tests to assess associations between Centor score, RADT results, and age groups. A p-value of <0.05 was deemed statistically significant.

## Results

Patient demographics

A total of 103 patients were included, with a slight male preponderance of 56 (55%). Median age was two years, with 57% (N=59) of patients being under three years old. Nearly half 45 (43.7%) had siblings, and two-thirds 67 (65%) attended childcare.

Documentation

**Figure 1:**
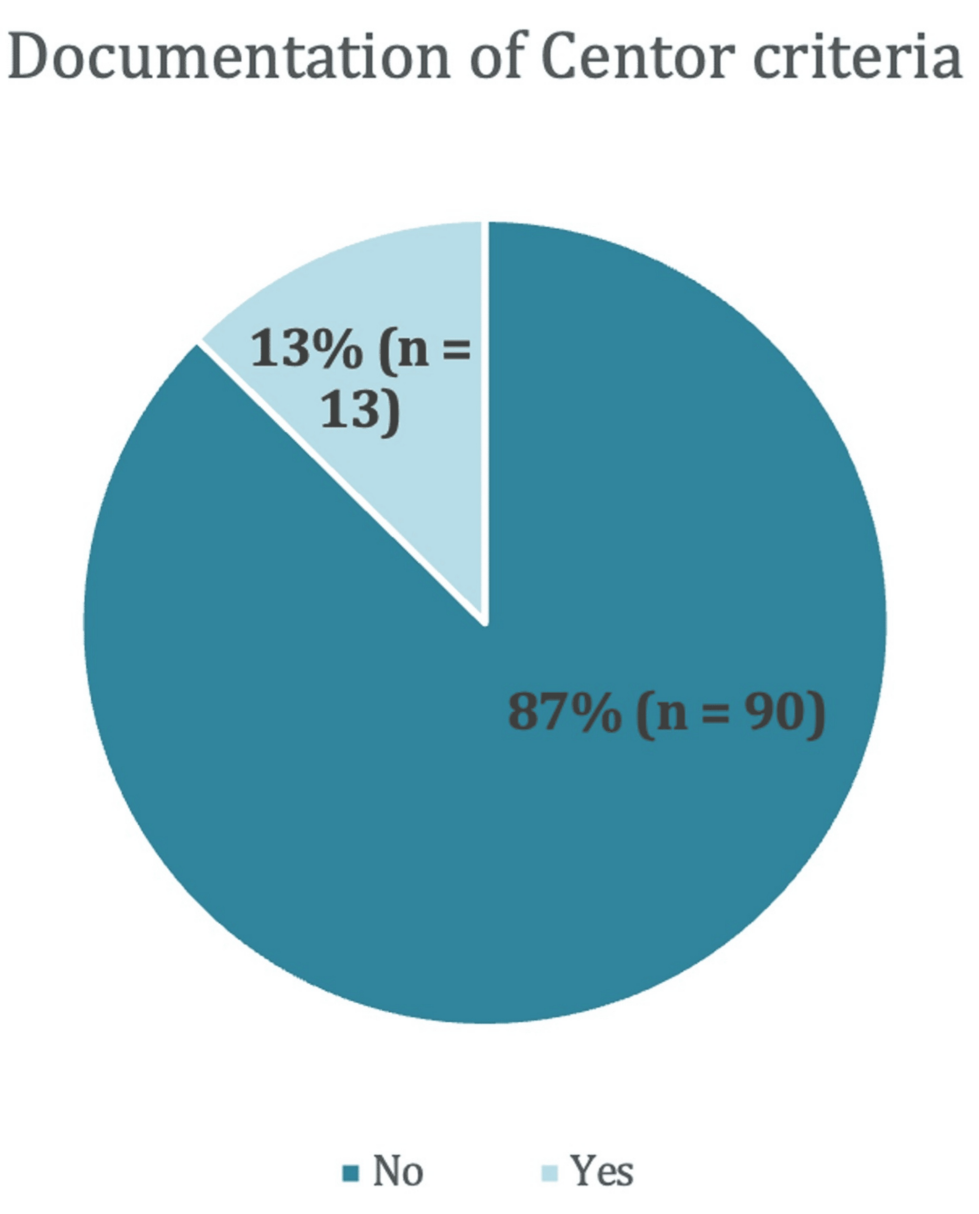
Documentation of Centor criteria. The data have been represented as N and %.

Centor criteria were not documented for the majority of patients, as shown in Figure 1.

**Figure 2:**
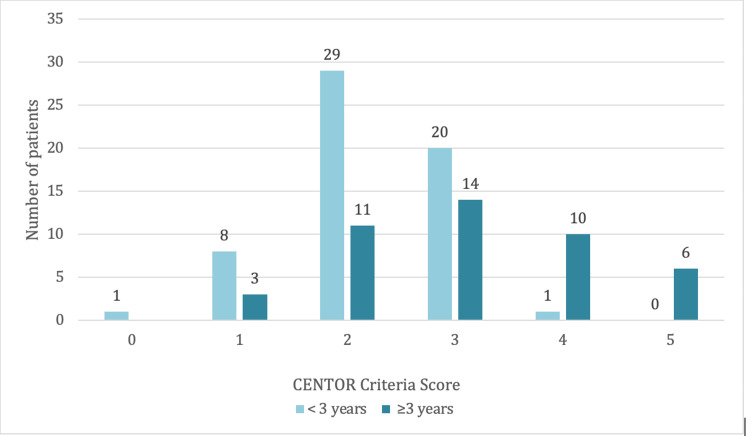
Centor score according to patients' age. Data have been represented as N.

Figure 2 shows the Centor scores across the different age groups, with the most common Centor score being two in children <3 years and three in those ≥3 years.

Overall, only 49.5% (N=53) had a Centor score of ≥3 (Table 1), with children aged ≥3 years being significantly more likely to achieve this score (p=0.001).

**Table 1:** Age group vs Centor criteria score. Data have been represented as N and %. A p-value of <0.05 was considered statistically significant. The chi-square test was used to calculate the p-value. The chi-square value is 10.71 and df=1.

Age group	Centor ≥3 n (%)	Centor <3 n (%)	Chi-square (p-value)
<3 years	21 (36%)	38 (64%)	0.001
≥3 years	30 (68%)	14 (32%)	

Rapid antigen detection test (RADT) performance

RADT was performed in all patients (100%, N=103) while no throat cultures were sent. This may have affected the accuracy of the RADT-negative swabs, especially as in older children, this should not be the first-line test according to guidelines. Thirty-one patients (30%) tested positive. Table 2 shows the geographic distribution of these patients.

**Table 2:** Distribution of patients with a positive GAS RADT according to the region. The data have been represented as N and %. GAS: group A streptococcus; RADT: rapid antigen detection test.

Region (Malta)	Count n (%)
North	8 (26%)
Central	23 (74%)
South	0 (0%)
Total	31 (100%)

There was a statistically significant association between age group and group A strep throat swab result, as shown in Table 3. Children ≥3 years old were significantly more likely to test positive when compared to the younger age group (41% (N=18) vs 16% (N=9), p < 0.004).

**Table 3:** GAS swab results according to age. Data have been represented as N and %. A p-value of <0.05 was considered statistically significant. The chi-square test was used to calculate the p-value. The chi-square value is 8.29 and a df=1. GAS: group A streptococcus; RADT: rapid antigen detection test.

GAS swab	GAS RADT positive n (%)	GAS RADT negative n (%)	Chi-square (P-value)
<3 years	9 (16%)	49 (84%)	0.004
≥3 years	18 (41%)	26 (59%)	

Treatment

All patients with positive RADT results were prescribed antibiotics, with 88% (N=23) receiving a ten-day course of co-amoxiclav. Cephalosporins were prescribed for two (2%) patients, and one (1%) patient received clarithromycin in view of penicillin allergy.

Notably, 32% (N=24) of patients with a negative RADTs (73%, N=75) were also prescribed antibiotics, most commonly amoxicillin, contrary to guideline recommendations. Among RADT-negative children, those with Centor scores ≥3 were more likely to be prescribed antibiotic treatment.

## Discussion

This study provides a unique perspective on the use of RADTs for GAS pharyngitis among pediatric patients presenting to the single pediatric emergency department within Malta's national health scheme. Gaps in adherence to the Centor score and excess antibiotic prescribing have been identified.

Demographics and epidemiology

Our cohort had a median age of two years. Nearly half the patients were under three years old, reflecting a younger age group than many international studies involving school-aged children. The overall RADT positivity rate was 30% (N=31), which compares well with the pooled prevalence estimate of 37% reported by Shaikh et al. for children presenting with pharyngitis [11]. In our cohort, there were significantly higher rate of positive Centor scores, i.e., ≥3 (68% (N=70) vs 36% (N=37)) and positive RADT tests in children ≥3 years old (41% (N=42) vs 16% (N=17)), in keeping with this age-related trend and highlighting the value of testing school-aged children, who have a higher burden of disease.

Documentation and application of the clinical scoring system

Centor criteria were not documented in 87% (N=90) of patients, but only half of the patients, 52 (50.5%), tested met the threshold score of ≥3, with the remaining patients being tested outside guideline recommendations. These findings are similar to those by Flamant et al. [12] in their prospective observational study in a large pediatric emergency department, where RADTs were not indicated in 66% (N=68) of the patient cohort. This reflects a lack of consistency in applying and following the criteria for testing and demonstrates a tendency toward over-investigation of patients.

Investigations

The positive predictive value of patients with a Centor score ≥3 having a positive GAS RADT was about 33%. This low value is in line with international evidence. Kanagasabai et al. [13], in a meta-analysis, found insufficient sensitivity and specificity of the Centor and McIsaac scores for them to be used as the sole indicator for antibiotic treatment without confirmatory testing. Le Marechal et al. [5], in an earlier meta-analysis, also concluded that no symptom or scoring system in isolation could reliably rule in GAS pharyngitis.

Antibiotic prescribing patterns

All patients with positive RADT results (30%, N=31) were prescribed antibiotics, with co-amoxiclav prescribed in most cases, although the guidelines suggest penicillin or amoxicillin as first line. Locally, penicillin and amoxicillin are not readily available as syrups in the community, leading to the frequent prescription of co-amoxiclav. Notably, a significant proportion (32%, N=23) of patients with a negative RADT were also prescribed antibiotics, contrary to guideline recommendations. This trend in antibiotic prescribing was also noted in other international studies in general practice and in emergency departments [12,14].

Among patients with a negative GAS swab, 34 (47%) had a Centor score ≥3, but only 13 (38%) of them were prescribed antibiotics. This suggests that physicians relied on the GAS RADT result rather than the clinical score to guide treatment.

Strengths and limitations

This study is a retrospective nationwide analysis. It was carried out across two seasons during a period of increased global prevalence of GAS infections, making it both timely and medically relevant. To our knowledge, it is the first study of its kind conducted in Malta, contributing valuable local data to the broader literature.

However, the study has several limitations. The sample size was relatively small and skewed towards younger children. Data collection relied on physicians reporting any swabs taken, with potential inaccuracies or underreporting. Documentation inconsistencies also posed a challenge, particularly for clinical scores, with Centor scores recorded in only 12.9% (N=13) of patients.

## Conclusions

Although RADTs for GAS are readily available for use and positive results prompt appropriate antibiotic prescribing, this study highlights that there are ongoing areas for improvement in the application of clinical criteria, indicating the need for testing and in antimicrobial stewardship. The overuse of RADT and the prescription of antibiotics for children whose RADT is negative emphasize the need for better documentation of the Centor score, improved adherence to the testing algorithm (including the Centor score), and the use of culture confirmation for patients with a high clinical suspicion of GAS pharyngitis in spite of negative RADT. These findings are similar to international recommendations and highlight the need for more consistent application of clinical guidelines and antimicrobial stewardship.
